# Facile Synthesis of Spherical TiO_2_ Hollow Nanospheres with a Diameter of 150 nm for High-Performance Mesoporous Perovskite Solar Cells

**DOI:** 10.3390/ma14030629

**Published:** 2021-01-29

**Authors:** Hoang Van Quy, Dang Hai Truyen, Sangmo Kim, Chung Wung Bark

**Affiliations:** Department of Electrical Engineering, Gachon University, Seongnam 13120, Korea; quybk@gachon.ac.kr (H.V.Q.); danghaitruyen@gmail.com (D.H.T.); singmul0227@gachon.ac.kr (S.K.)

**Keywords:** perovskite solar cells, hollow nanosphere, ETLs

## Abstract

The electron transport layer (ETL) of organic–inorganic perovskite solar cells plays an important role in their power conversion efficiency (PCE). In this study, TiO_2_ hollow nanospheres with a diameter of 150 nm were prepared by a facile synthesis method. The synthesized TiO_2_ hollow nanospheres had a highly porous structure with a surface area of 85.23 m^2^ g^−1^, which is significantly higher than commercial TiO_2_ (P25) (54.32 m^2^ g^−1^), indicating that they can form an ideal mesoporous layer for Formamidinium iodide-based perovskite solar cells (PSCs). In addition, the nanospheres achieved a remarkable perovskite performance, and the average PCE increased from 12.87% to 14.27% with a short circuit current density of 22.36 mAcm^−2^, an open voltage of 0.95 V, and a fill factor of 0.65. The scanning electron microscopy images revealed that the enhanced PCE could be due to the improved carrier collection and transport properties of the nanosphere, which enabled efficient filtration of perovskite into the TiO_2_ mesoporous ETL. The TiO_2_ hollow nanospheres fabricated in this study show high potential as a high-quality ETL material for efficient (FAPbI_3_)_0.97_(MAPbBr_3_)_0.03_-based PSCs.

## 1. Introduction

Hybrid organic-inorganic perovskite solar cells (PSCs) that employ formamidinium lead halide as a light-absorbing material have been applied to various photovoltaic devices owing to their excellent optoelectronic properties [1,2,3,4,5,6,7]. Over the years, the photovoltaic conversion efficiency (PCE) of PSCs has been improved to obtain efficient and affordable PSC devices, with the PCE recently reaching 25.2% [8]. In mesoscopic PSCs, the perovskite is prepared on a mesoporous TiO_2_ layer, which accepts photoexcited electrons from the absorbers and transports them to the fluorine-doped tin oxide (FTO) substrate. Mesoporous layer-free PSCs have been demonstrated to exhibit high PCE in planar-structured devices; however, planar-structured devices show hysteresis behavior and trap the photogenerated free electrons, thus hindering further enhancement of the PCE [9,10,11,12,13,14,15]. Therefore, it is important to develop ideal mesoporous materials that possess sizeable pores, extended contact areas, and defect-free nanostructures with negligible boundaries to prevent the charge recombination [16,17,18]. Small-sized TiO_2_ nanoparticles (NPs) possess a large surface area; however, their narrow pore size reduces the infiltration of the perovskite absorber to the electron transport layer (ETL) [19,20,21]. In contrast, larger-sized TiO_2_ NPs possess a smaller surface area, which affects efficient charge injection through the hole transport layer (HTL). Therefore, it is important to develop an optimized mesoporous material with relatively large TiO_2_ NPs to form a scaffold structure for the collection of electrons from the perovskite layer.

Hui Wang et al. [22] fabricated hollow TiO_2_ nanospheres with a shell thickness of approximately 30 nm and applied them in PSCs. The PSC achieved a PCE of 15.87%, which could be due to the improved carrier transport properties and matching of the hollow TiO_2_ to the perovskite layer. In addition, hollow spheres TiO_2_ with a radius of 200–300 nm using titanium isopropoxide as precursor material were fabricated in a previous study. The TiO_2_ was applied in a PSC, and the PSC achieved a PCE of 14.2% owing to the suppression of carrier recombination at perovskite/TiO_2_ interfaces [23]. These results indicate that the PCE of PSCs can be further enhanced using hollow TiO_2_ nanospheres with a uniform shape as the mesoporous ETL.

In this study, a large spherical TiO_2_ hollow structure with a diameter of 150 nm was successfully synthesized by controlling the sol-gel reaction. The synthesized nanoporous spherical TiO_2_ hollow nanospheres was a monodispersed material with a porous structure and high surface area and was thus highly suitable for fabricating mesoporous PSCs. The PSC fabricated with the as-synthesized TiO_2_ hollow nanospheres exhibited enhanced photovoltaic properties, and the PCE increased from 12.87% to 14.27% under AM 1.5G irradiation (100 mW·cm^−2^).

## 2. Materials and Methods

### 2.1. Synthesis of Carbonaceous Nanospheres (CNs)

Carbonaceous nanospheres (CNs) were synthesized according to the method developed by Sun et al. and reported in other researches [24,25]. Briefly, 89 g of glucose (Sigma-Aldrich, St. Louis, MO, USA) was firstly dissolved in water (250 mL) to form a clear solution, and the solution was transferred into a 500 mL Teflon autoclave and heated at 180 °C for 5.5 h. The mixture was collected by centrifugation, washed 3–4 times with ethanol and water, and oven-dried at 80 °C for over 4 h for further experiments.

### 2.2. Synthesis of the TiO_2_ Hollow Nanospheres

To synthesize the mesostructured TiO_2_ hollow nanospheres, 0.02 g of the as-synthesized CNs was homogeneously dispersed in ethanol (10 mL) by ultrasonication and stirred for 30 min at room temperature. Then, 0.04 mL of titanium isopropoxide (TIIP) (Sigma-Aldrich) was slowly injected into the dispersion under vigorous stirring for 30 min. CNs-TiO_2_ nanospheres were collected by centrifugation; they were washed with a mixture of ethanol/water several times and then dried in air for a day. Finally, the as-synthesized CNs-TiO_2_ nanospheres were sintered at 450 °C for 2 h in a muffle furnace under air to obtain the TiO_2_ hollow nanospheres.

### 2.3. Fabrication of the Perovskite Solar Cells

Briefly, a glass/FTO substrate (the sheet resistance of 15 Ωsq^−1^) was cleaned with isopropanol, acetone, distilled water, and ethanol. The cleaned FTO substrate was dried under a N_2_ stream and treated with ultraviolet ozone for 15 min to remove any organic contamination. To prepare the compact TiO_2_ layer on the substrate, the compact TiO_2_ solution was spin-coated on the cleaned substrate using a titanium diisopropoxide bis(acetylacetone) (75% in 1-butanol) solution (0.15 M, 2000 rpm, 20 s), after which the substrate was dried at 125 °C for 20 min. The substrate was cooled down to room temperature, and the porous layer was spin-coated on the compact TiO_2_ at 4000 rpm for 20 s using the TiO_2_ paste (Supplementary Materials Figure S1) diluted in ethanol (1:6, wt:wt), and finally annealed at 480 °C for 30 min. After cooling down to room temperature, the samples were moved into a nitrogen-filled glovebox (water and oxygen content below 1 ppm) for fabricating perovskite and HTL layers. The perovskite layer was deposited by a two-step deposition method. First, the PbI_2_ precursor solution was spin-coated onto the ETL at 2000 rpm for 20 s using 1.3 M PbI_2_ (600 mg PbI_2_ in 900 μL of N, N-Dimethylformamide (DMF, Sigma-Aldrich) and 100 μL of Dimethyl sulfoxide (DMSO, Sigma-Aldrich). Subsequently, a mixture perovskite precursor solution of FAI:MABr:MACl (60:6:6, mg) in 1 mL of 2-propanol was loaded onto the PbI_2_ layer for 20 s (loading time) and then spin-coated at 4000 rpm for 20 s. The perovskite films were heated at 150 °C for 15 min. Next, a solution containing 72.3 mg of spiro-MeOTAD (Sigma-Aldrich), 1 mL of chlorobenzene (Sigma-Aldrich), 28.8 μL of 4-*tert*-butylpyridine, and 17.5 μL of Li-TFSI solution (52 mg Bis(trifluoromethane)sulfonimide lithium salt (Li-TSFI, Sigma-Aldrich) in 100 μL acetonitrile (Sigma-Aldrich) was deposited on top of the perovskite layer. Finally, 80 nm gold electrodes were thermally evaporated on the spiro-MeOTAD film.

### 2.4. Device Characterization

The ultraviolet-visible (UV-vis) light absorption spectra of the films were measured using a UV-vis spectrophotometer (Agilent 8453, Agilent technologies, Santa Clara, CA, USA) to evaluate the absorption property of the films. The X-ray diffraction (XRD) patterns of the samples were obtained using a XRD Rigaku DMAX 2200 system (Rigaku, Tokyo, Japan) with Cu Kα (λ = 0.15406 nm) as the X-ray source. The specific Brunauer–Emmett–Teller (BET) surface areas of the samples were investigated using an ASAP 2020 (Micromeritics, Atlanta, GA, USA) apparatus. An infrared spectrometric analyzer (Vertex 70, Bruker, Ettlingen, Germany) was used to record the Fourier transform infrared (FTIR) spectra. The top and cross-sectional morphologies of the samples were examined by field emission scanning electron microscopy (FESEM, Hitachi S-4700, Tokyo, Japan) operated at 10 kV. The steady-state photoluminescence (PL) spectra were determined using a QuantaMaster TM 50 PTI (Birmingham, New Jersey, NJ, USA). A sun simulator (Polaromix K201, Solar simulator LAB 50, McScience K3000, McScience, Gyeonggi-do, Korea) with an irradiance of 100 mW cm^−2^ (AM 1.5G) was used to simulate solar irradiation. The external quantum efficiency (EQE) was measured using McScience K3100 measurement system (McScience, Gyeonggi-do, Korea).

## 3. Results

**Figure 1:**
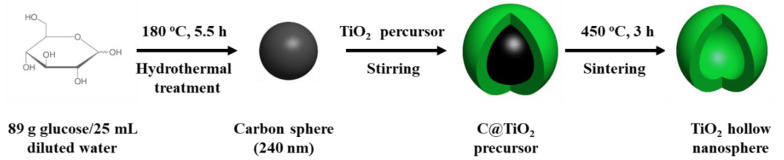
Schematic illustration of the synthesis procedure of spherical carbon nanoparticles (NPs).

In this study, to synthesize the TiO_2_ hollow nanospheres, uniform CNs (diameter of around 240 nm, Supplementary Materials Figure S2) were synthesized by a hydrothermal method using glucose, which is easily disintegrated at low temperatures (under 450 °C), as the soft template. The schematic of the synthesis procedure is shown in Figure 1. Particularly, a titanium isoporoxide aqueous solution was used as the precursor solution during the reaction and was absorbed by the negatively charged CNs.

**Figure 2:**
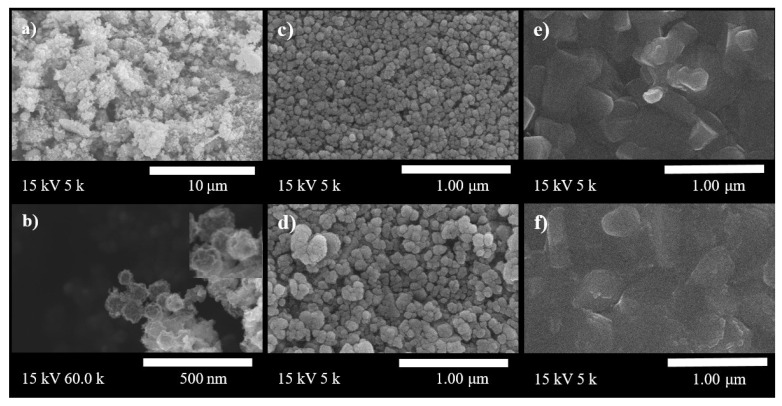
(**a**,**b**) SEM image of the P25 and TiO_2_ hollow powder. (**c**,**d**) SEM images of commercial TiO_2_ (P25) paste and the hollow TiO_2_ paste. (**e**,**f**) SEM images of the FTO/cpTiO_2_/P25 (hollow TiO_2_)/perovskite.

The SEM images of the P25 TiO_2_ powders (Sigma-Aldrich).and hollow nanospheres are shown in Figure 2a,b. It can be seen that samples nanospheres in diameter were estimated to be ~150 nm; they were monodispersed during the entire synthesis process, while P25 are tiny with aggregated particles. The effect of the size of the TiO_2_ hollow nanosphered and P25 on the ETL was examined. The surface morphologies of P25 and the as-synthesized TiO_2_ hollow nanospheres (Figure 2c,d) were analyzed by FESEM. As shown in the image, both TiO_2_ nanoparticles are uniformly distributed in the TiO_2_ films. However, the size of the TiO_2_ hollow nanospheres was significantly larger than that of the P25. Figure 2e,f shows the surface morphologies of the perovskite layer on the mesoporous TiO_2_ films. The perovskite deposited on the TiO_2_ hollow nanospheres exhibited a smooth and flattened surface morphology with fewer pinholes than that deposited on the P25.

**Figure 3:**
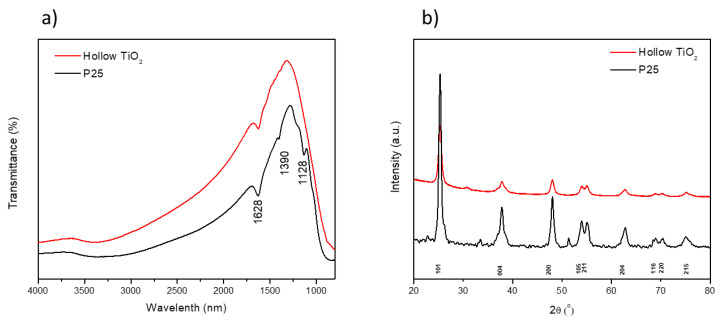
(**a**) FTIR of P25 powder and TiO_2_ hollow powder. (**b**) XRD patterns for the P25 and TiO_2_ hollow powders.

Figure 3a shows the FTIR spectra of the P25 and TiO_2_ hollow nanospheres recorded from 4000 cm^−1^ to 400 cm^−1^. The peaks at 3650 and 1628 cm^−1^ were ascribed to the C–H stretching region and H–O–H bending vibrations signature of water molecules. In addition, a strong absorption peak of Ti–O–Ti vibration was observed at 500 cm^−1^ in both samples, indicating that there was no shift in the main peak of Ti–O bonding compared to the TiO_2_ anatase phase of P25. Additionally, the characteristic peak of the C–H stretching vibration at 2920 cm^−1^ was not observed, confirming that the carbon nanosphere template was completely removed during the calcination process.

The crystal structure of the obtained powder was further investigated by XRD. The XRD patterns of P25 and the TiO_2_ hollow nanospheres were recorded from 20 to 80°, as shown in Figure 3b. The XRD peaks at 25.2°, 37.8°, 48.1°, 53.9°, 55.1°, 62.8°, 68.9°, 70.3°, and 75.1° were ascribed for the TiO_2_ anatase crystalline structure, which is consistent with the ICSD, No. 75-1537. In addition, the carbon reflection peak was not observed in the sample after calcination, which is consistent with the FTIR results [26]. Furthermore, the crystalline size of the TiO_2_ hollow nanospheres determined using the Debye-Scherrer equation from anatase (101) was approximately 11 nm.

**Figure 4:**
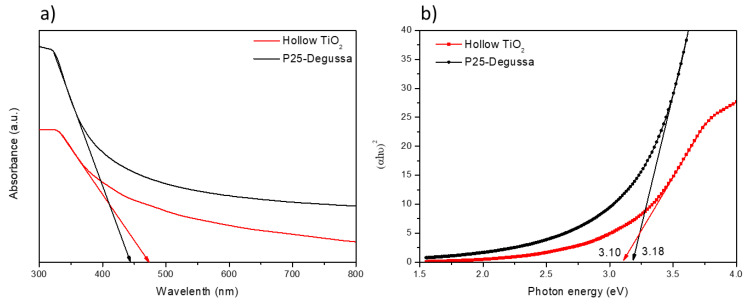
(**a**) UV-vis absorbance spectra. (**b**) Band gap energy of the P25 and TiO_2_ hollow nanospheres.

The optical properties of the P25 and TiO_2_ hollow nanospheres were investigated by UV-vis spectrophotometry in the range of 300–800 nm (Figure 4a). Their direct bandgap energy was estimated by plotting (αhν)^2^ versus the photon energy, Eg = 1240/λ, as shown in Figure 4b. As shown in the image, the bandgap energies of the P25 and TiO_2_ hollow nanospheres were 3.18 and 3.10 eV, respectively. The low bandgap energy of the hollow TiO_2_ nanospheres could be due to the higher average crystal size of the TiO_2_ hollow nanospheres because of the quantum size-effect [27,28]. Therefore, the decrease in the bandgap between the conduction band and the valence band was expected to enhance the charge injection from the perovskite layer to the ETL.

**Figure 5:**
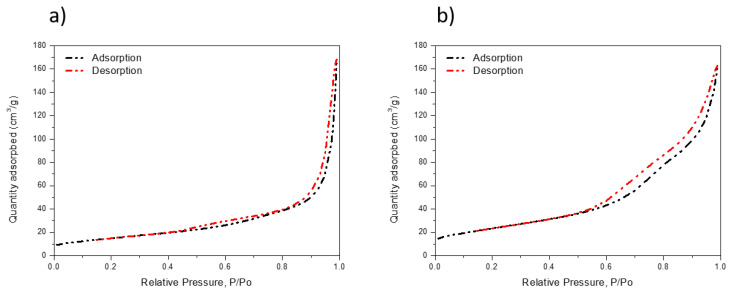
N_2_ adsorption-desorption isotherms of (**a**) P25 and (**b**) TiO_2_ hollow nanospheres.

The specific surface areas and pore size distributions of the P25 and TiO_2_ hollow nanospheres were investigated by N_2_ adsorption/deposition analysis, and the results are shown in Figure 5a,b. The TiO_2_ hollow nanospheres exhibited a broad hysteresis loop of type IV isotherms at a relative pressure of 0.5, showing that it is a mesoporous structure. However, the TiO_2_ hollow nanospheres exhibited a relatively narrow pore size distribution of approximately 11.24 nm. In addition, the TiO_2_ hollow nanospheres had a large surface area of 85.23 m^2^g^−1^, which was 1.56 times higher than that of P25 (54.32 m^2^g^−1^). The higher surface area of the hollow TiO_2_ nanospheres is expected to facilitate the efficient filtration of perovskite to the ETL, thus improving the photovoltaic properties of the PSC.

**Figure 6:**
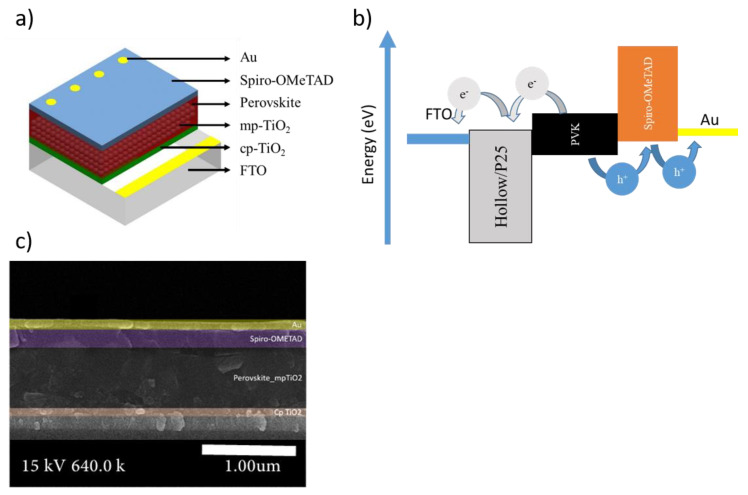
(**a**) The schematic of the device structure perovskite solar cells (PSCs) based on TiO_2_ hollow nanospheres. (**b**) The schematic diagram of band alignment in the complete device. (**c**) Cross-sectional image of the PSC device.

The commercial TiO_2_ and TiO_2_ hollow nanospheres were employed as ETLs in the mesoporous-structured perovskite solar cells consisting of FTO/c-TiO_2_/mp-TiO_2_/perovskite/Spiro/Au (Figure 6a). Figure 6b indicates an energy diagram of the PSCs with P25 and TiO_2_ hollow nanospheres. The cross-section image of the PSC fabricated using the TiO_2_ hollow nanospheres (Figure 6c) revealed that the thicknesses of the Spiro-OMeTAD HTL and Au electrodes were approximately 250 nm and 80 nm, respectively. The enhanced physical properties of the TiO_2_ hollow nanospheres are summarized in Table 1.

**Figure 7:**
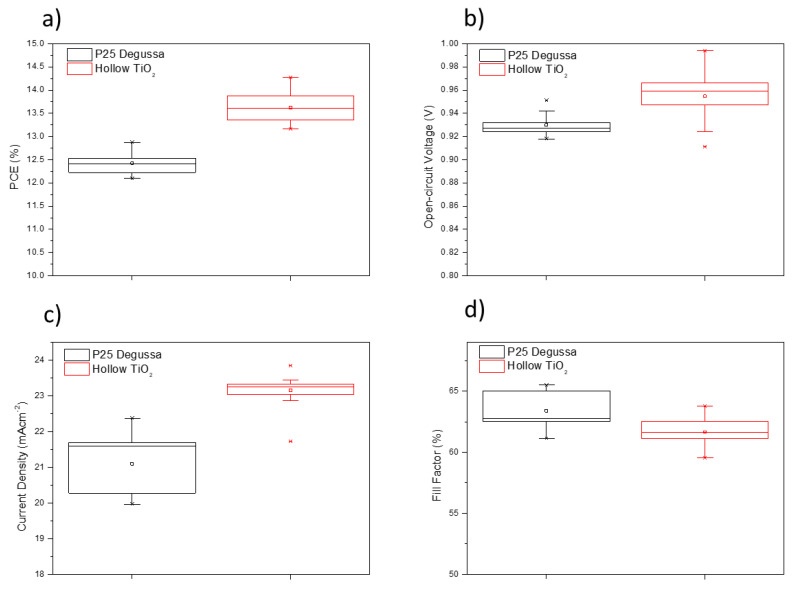
Box chart of the PSC photovoltaic parameters based on P25 and as-synthesized hollow TiO_2_ nanospheres. The data was collected from 18 cells for each type. (**a**) PCE (%); (**b**) short circuit current density (*J*_SC_) (mAcm^−2^); (**c**) fill factor (FF); (**d**) V_OC_ (V).

To investigate the applicability of the TiO_2_ hollow nanospheres as an efficient ETL for PSC, the perovskite layer of (FAPbI_3_)_0.97_(MAPbBr_3_)_0.03_ was fabricated by a two-step deposition method [4,29,30]. Thirty-six independent PSCs fabricated using P25 and TiO_2_ nanospheres as the ETLs were examined, the results are shown in Figure 7a–d; the corresponding champion and average values are summarized in Table 2. The P25-based mesoporous layer champion device exhibited a PCE of 12.87% with a short circuit current density (*J*_SC_) of 22.369 mAcm^−2^, an open voltage (V_OC_) of 0.951 V, and a fill factor (FF) of 0.65. In contrast, the TiO_2_ hollow nanosphere-based champion device exhibited an enhanced PCE of 14.27% with a *J*_SC_ of 23.84 mAcm^−2^, V_OC_ of 0.994, and FF of 0.64, with excellent reproducibility. The remarkable increase in *J*_SC_ and V_OC_ could be due to the significantly enhanced optical and morphological properties of the TiO_2_ hollow nanospheres.

**Figure 8:**
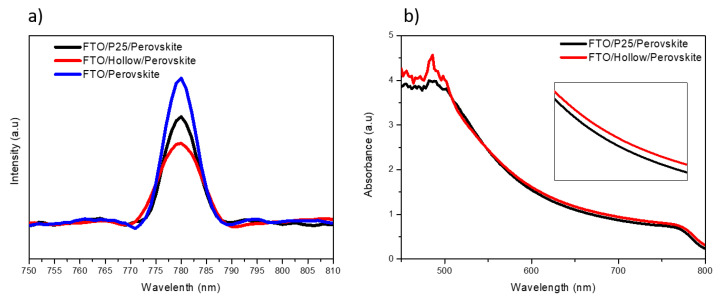
(**a**) PL spectra of the P25 and TiO_2_ hollow nanosphere-based perovskite films. (**b**) Absorbance spectra of perovskite deposited on P25 and hollow TiO_2_ layers.

The PL of the samples was measured and analyzed to investigate the charge extraction dynamics at the perovskite/ETL interface. Figure 8a shows the PL spectra of the FTO/perovskite, FTO/cp-TiO_2_/P25/perovskite, and FTO/cp-TiO_2_/TiO_2_ hollow nanospheres/perovskite at wavelengths from 750 to 810 nm at a light excitation of 530 nm, where the emission peaks were obtained at approximately 780 nm for all the films. The PL intensity peak of the TiO_2_ hollow nanospheres decreased significantly compared to that of the P25 sample, which could be due to the efficient filtration of the perovskite precursor into the mesoporous structure and the reduced charge recombination behavior at the perovskite/ETL interfaces. As shown in Figure 8b, in comparison to the absorbance in the perovskite deposited on P25 and hollow TiO_2_ samples, an enhanced absorption value of perovskite/hollow TiO_2_/TFO sample from 550 nm to 800 nm can be realized for the efficient absorber layer of PSCs.

**Figure 9:**
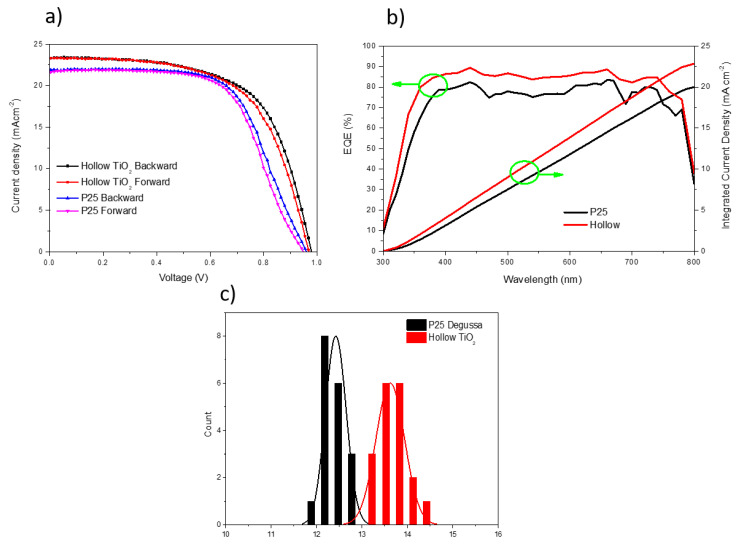
(**a**) *J*-V curves of the P25 and TiO_2_ hollow nanosphere-based PSCs. (**b**) External quantum efficiency of the P25 (hollow)-based devices and integrated current density. (**c**) PCE distributions of the 36 cells of the P25 and TiO_2_ hollow nanosphere-based devices.

Because the mesoporous scaffold plays an important role in the recombination behavior and hysteresis effect of PSCs, the current-voltage (*J*-V) curves of the forward and backward scans were obtained to investigate the hysteresis of the ETL-based PSCs (Figure 9a) [31,32,33]. The external efficiency quantum of the complete devices is shown in Figure 9b. The integrated current density of the TiO_2_ hollow nanospheres is 23 mAcm^−2^ with the external quantum efficiency of 89.5%, which is higher than that of P25. The high *J*_SC_ could be due to the high quality of perovskite layer loaded on the TiO_2_ hollow nanospheres.

As shown in the image, the TiO_2_ hollow nanospheres exhibit relatively less hysteresis in the *J*-V measurement than P25. Furthermore, the TiO_2_ hollow nanosphere-based PSCs exhibited a high reproductivity with the average PCEs of 18 cells in the range from 13.5 to 14.5% (Figure 9c). In conclusion, the TiO_2_ hollow nanospheres with a diameter of 150 nm exhibited highly specific surface areas, thus making them ideal ETL materials for fabricating high-efficiency PSCs. The photovoltaic performance of the PSCs based on hollow TiO_2_ and P25 in this study ware compared with those of the PSCs reported in previous studies (see Table 3).

## 4. Conclusions

In conclusion, TiO_2_ hollow nanospheres with a diameter of 150 nm were fabricated by a simple and effective method and applied as mesoporous ETLs for PSCs. The fabricated PSCs based on the TiO_2_ hollow nanospheres exhibited a PCE of 14.27% under AM 1.5G irradiation (100 mW·cm^−2^) with a *J*_SC_ of 23.84 mAcm^−2^, V_OC_ of 0.94 V, and FF of 0.64, which were higher than those of the P25-based PSCs. In addition, the PL measurement revealed efficient electron injection from the perovskite to the TiO_2_ layer, which could be due to the increase in the pore size of the hollow nanospheres.

## Figures and Tables

**Table 1:** Physical properties of P25 and TiO_2_ hollow nanospheres.

TiO_2_	Diameter (nm)	Bandgap (eV)	Surface Area (m^2^g^−1^)	Average Pore Size (nm)
P25	<25	3.18	54.32	15.81
TiO_2_ hollow	~150	3.10	85.23	11.24

**Table 2:** The photovoltaic performances of the PSCs under sunlight illumination of 100 mWcm^−2^ (AM 1.5 G). The results are based on the average values of 36 cells.

PSCs		*J*_SC_ (mAcm^−2^)	V_OC_ (V)	FF	PCE (%)
P25	Champion	22.369	0.951	0.65	12.87
	Average	21.097 ± 0.068	0.930 ± 0.021	0.63 ± 0.021	12.42 ± 0.45
TiO_2_ hollow	Champion	23.842	0.994	0.64	14.27
	Average	23.149 ± 0.693	0.955 ± 0.039	0.61 ± 0.021	13.62 ± 0.646

**Table 3:** Comparison of the photovoltaic parameters of recently PSCs based on hollow TiO_2_ nanospheres and commercial TiO_2_ (P25).

Particle Size (nm)	Structural Type	Preparation Method	*J*_SC_ (mAcm^−2^)	V_OC_ (V)	FF	PCE (%)	Ref.
150 nm	Hollow nanospheres	Sol-gel reaction	23.84	0.99	0.64	14.27	This study
200 nm	Hollow nanospheres	Sol-gel reaction	22.23	1.07	0.74	17.60	[34]
250 nm	Hollow nanospheres	Sol-gel reaction	23.92	1.01	0.65	15.87	[22]
300 nm	Hollow rice grain-shaped	Electro-spinning	21.60	1.07	0.61	4.20	[23]
100 nm	Spherical aggregates	Hydrothermal reaction	22.91	1.04	0.75	18.41	[16]
250 nm	Spherical aggregates	Sol-gel reaction	19.41	1.05	0.73	15.01	[35]
<25 nm	Nanoparticles	Commercial	20.83	0.89	0.67	12.48	[36]
<25 nm	Nanoparticles	Commercial	20.3	974.1	0.71	14.1	[37]
<25 nm	Nanoparticles	Commercial	22.369	0.951	0.65	12.87	This study

## Data Availability

Data is contained within the article or supplementary material.

## Supplementary Materials

The following are available online at https://www.mdpi.com/1996-1944/14/3/629/s1, Figure S1: Schematic representation for fabrication of TiO_2_ paste, Figure S2: SEM image of carbonaous nanosphere prepared by hydrothermal reaction.
