# Supplementary material for: Facile Synthesis of Spherical TiO2 Hollow Nanospheres with a Diameter of 150 nm for High-Performance Mesoporous Perovskite Solar Cells
Source: Materials (Basel). 2021 Jan 29;14(3):629. doi: 10.3390/ma14030629 (PMC7866397; doi:10.3390/ma14030629)
Supplement: Supplementary file 1 [file materials-14-00629-s001.zip › materials-1074516-supplementary.pdf]

materials

MDPI

SUPPLEMENTARY

Facile Synthesis of Spherical TiO_2 Hollow Nanospheres with a Diameter of 150 nm for High-Performance Mesoporous Perovskite Solar Cells

Hoang Van Quy, Dang Hai Truyen, Sangmo Kim and Chung Wung Bark *

Department of Electrical Engineering, Gachon University, Seongnam, Korea; quybk@gachon.ac.kr (H.V.Q); danghaitruyen@gmail.com (D.H.T.); singmul0227@gachon.ac.kr (S.K.)
* Correspondence: bark@gachon.ac.kr

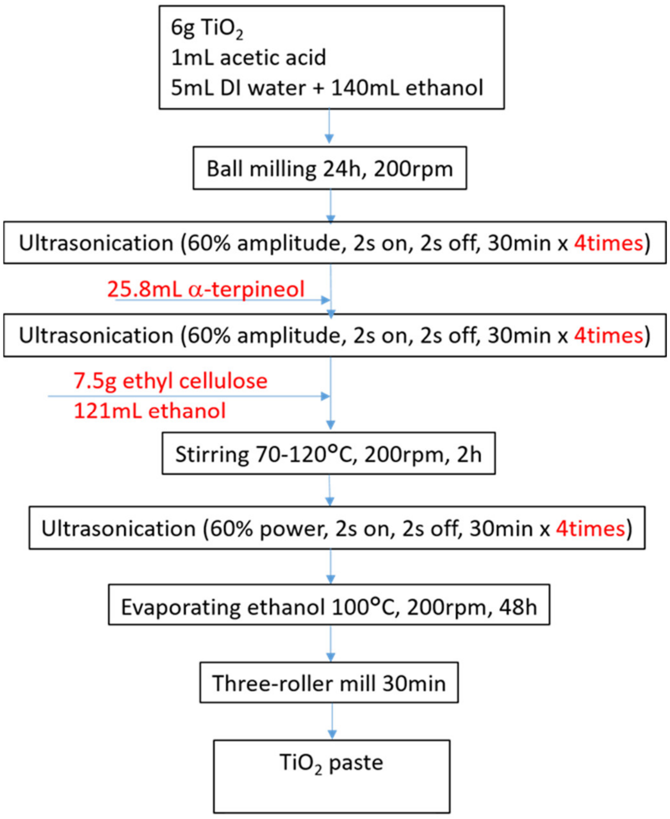

Figure S1. Schematic representation for fabrication of TiO_2 paste.

Citation: Quy, V.H.; Truyen, D.H.; Kim, S.; Bark, C.W. Facile synthesis of spherical TiO_2 hollow nanospheres with a diameter of 150 nm for high-performance mesoporous perovskite solar cells. **2021**, *14*, 629. https://doi.org/10.3390/ma14030629

Received: 29 December 2020
Accepted: 27 January 2021
Published: 29 January 2021

Publisher's Note: MDPI stays neutral with regard to jurisdictional claims in published maps and institutional affiliations.

Copyright: © 2021 by the authors. Licensee MDPI, Basel, Switzerland. This article is an open access article distributed under the terms and conditions of the Creative Commons Attribution (CC BY) license (http://creativecommons.org/licenses/by/4.0/).

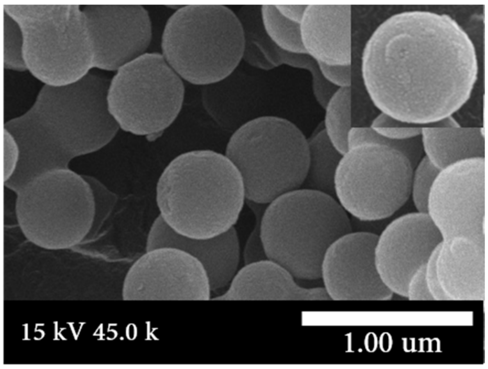

Figure S2. SEM image of carbonaous nanosphere prepared by hydrothermal reaction.
